# Zika Virus Infection in Pregnancy: Advanced Diagnostic Approaches in Dengue-Naive and Dengue-Experienced Pregnant Women and Possible Implication for Cross-Reactivity and Cross-Protection

**DOI:** 10.3390/microorganisms8010056

**Published:** 2019-12-28

**Authors:** Maurizio Zavattoni, Francesca Rovida, Elena Percivalle, Irene Cassaniti, Antonella Sarasini, Alessia Arossa, Beatrice Tassis, Lina Bollani, Giuseppina Lombardi, Simona Orcesi, Fausto Baldanti

**Affiliations:** 1 Molecular Virology Unit, Microbiology and Virology Department, Fondazione IRCCS Policlinico San Matteo, 27100 Pavia, Italy; m.zavattoni@smatteo.pv.it (M.Z.); f.rovida@smatteo.pv.it (F.R.); e.percivalle@smatteo.pv.it (E.P.); i.cassaniti@smatteo.pv.it (I.C.); a.sarasini@smatteo.pv.it (A.S.); 2 Departments of Obstetrics and Gynecology, Fondazione IRCCS Policlinico San Matteo, 27100 Pavia, Italy; alessia.arossa@yahoo.it; 3 Obstetrics and Gynecology Unit, Fondazione IRCCS Ca’ Granda Ospedale Maggiore Policlinico, 20122 Milan, Italy; beatrice.tassis@tin.it; 4 Neonatal Intensive Care Unit, Fondazione IRCCS Policlinico San Matteo, 27100 Pavia, Italy; l.bollani@smatteo.pv.it (L.B.); g.lombardi@smatteo.pv.it (G.L.); 5 Child Neurology and Psychiatry Unit, IRCCS Mondino Foundation, 27100 Pavia, Italy; simona.orcesi@mondino.it; 6 Section of Microbiology, Department of Clinical, Surgical, Diagnostic and Pediatric Sciences, University of Pavia, 27100 Pavia, Italy

**Keywords:** Zika virus, congenital infection, flavivirus, cross-reactivity, cross-protection

## Abstract

Zika virus (ZIKV) infection has been linked to congenital defects in fetuses and infants, as exemplified by the microcephaly epidemic in Brazil. Given the overlapping presence of Dengue virus (DENV) in the majority of ZIKV epidemic regions, advanced diagnostic approaches need to be evaluated to establish the role of pre-existing DENV immunity in ZIKV infection. From 2015 to 2017, five pregnant women with suspected ZIKV infection were investigated in Pavia, Italy. Among the five pregnant women, three were DENV–ZIKV immunologically cross-reactive, and two were DENV-naïve. Advanced diagnosis included the following: (i) NS1 blockade-of-binding (BOB) ELISA assay for ZIKV specific antibodies and (ii) ELISpot assay for the quantification of effector memory T cells for DENV and ZIKV. These novel assays allowed to distinguish between related flavivirus infections. The three DENV-experienced mothers did not transmit ZIKV to the fetus, while the two DENV-naive mothers transmitted ZIKV to the fetus. Pre-existing immunity in DENV experienced mothers might play a role in cross-protection**.**

## 1. Introduction

In 2015, the mosquito-borne Zika virus (ZIKV) began spreading throughout the Americas and clinicians reported unexpected increases in the numbers of babies born with microcephaly and of adults with Guillain-Barrè syndrome (GBS). A major finding in ZIKV vertical transmission was the relation between first trimester exposure and severe neurologic sequelae, such as ventriculomegaly and lissencephaly, which commonly aligns with microcephaly and cerebellar hypoplasia [1]. Brain tissue also showed evidence of tissue destruction—calcifications, gliosis, and necrosis [2]. Although transmission of ZIKV has declined in the Americas, outbreaks and infection clusters continue to occur in some regions, such as India and South-east Asia, where there are large populations of women of childbearing age who are susceptible to the virus [3].

Modeling of data from French Polynesia suggests about a 1% risk of microcephaly associated with maternal Zika virus infection in the first-trimester pregnancy, while a model based on data from a Zika outbreak in Bahia, Brazil, suggests a risk between 1% and 13% [4,5]. In U.S. territories (all U.S. states, the District of Columbia, and all U.S. territories except Puerto Rico), among women with timing of possible Zika infection exclusively during the first trimester, 11% had a fetus or infant with a birth defect [2]. Overall, in Brazil, a rate of 1.98 microcephaly cases per 10,000 live births per year was observed, representing an approximately nine-fold increase over the average prevalence during the previous 14 years [6,7]. In comparison, Colombia reported a smaller relative increase (four-fold), but the prevalence of reported microcephaly was approximately 9.6 per 10,000 live births [8], and in the USA, it was 8.8 per 10,000 live births [9]. There are several possible reasons for the differences in the microcephaly increase rate in the context of the ZIKV outbreaks in these countries. First, 50–75% of the population of Colombia resides at altitudes above 2000 m, in areas without mosquito vector (Aedes genus) circulation. On the other hand, the population at risk in the United States, although large, has significantly less exposure to the closely-related Dengue virus (DENV) than the Brazilian population affected by the Zika virus. This may have implications for the risk of fetal infection and disease [10,11]. ZIKV infection has been linked to congenital abnormalities in both women living in endemic countries and women living in non-endemic countries who travel to countries with active ZIKV outbreaks in early pregnancy. The effect of pre-existing immunity against flaviviruses on ZIKV infection outcomes—whether the immunity is elicited by infection or by immunization with flavivirus vaccines—is a matter of debate. Laboratory investigations have yielded contradictory findings with respect to whether DENV infection elicits an immune response that protects against ZIKV infection or exacerbates infection by way of antibody-dependent enhancement (ADE) [12,13]. Prospective studies in humans showed that prior dengue infection and pre-existing anti-DENV antibodies reduced, rather than enhanced, the risk of ZIKV infection and disease [14,15].

Owing to the co-circulation of both ZIKV and DENV in all countries involved in ZIKV outbreaks, traditional diagnostic approaches are hampered by flavivirus cross-reactivity and need new technologies able to discriminate specific antibody responses. In this report, we discuss diagnostic approaches in five cases of ZIKV infection in pregnancy and propose new advanced assays for the differential diagnosis between ZIKV and DENV infection in pregnant women. We also discuss the possible mechanisms of protection from vertical transmission and associated congenital abnormalities.

## 2. Patients and Methods

Samples were collected and stored between 2015 and 2017. All the experiments were performed on residual materials collected by clinicians and handled by our laboratory personnel, and data were analyzed anonymously according to a Regional Surveillance and Preparedness Plan (DGR12591, 27 December 2012). The retrospective analysis was performed according to the guidelines of the Institutional Review Board of the Fondazione IRCCS Policlinico San Matteo on the use of biologic specimens for scientific purpose in keeping with Italian law (art.13 D.Lgs 196/2003).

The diagnostic work-up included the following: (i) the determination of ZIKV IgM and IgG (anti-Zika virus ELISA (IgM) and anti-Zika virus ELISA (IgG) by Euroimmun, Lübeck, Germany); (ii) DENV 1–4 IgM and IgG antibodies (Dengue virus IgM Capture DXSelec^TM^ and Dengue virus IgG DxSelect^TM^ by Focus Diagnostics, Cypress, CA 90630, USA) in serum samples; along with (iii) confirmation of serological results by plaque reduction neutralization test (PRNT) for ZIKV and DENV 1–4 [16,17]; (iv) the determination of Chikungunya (CHKV) IgM and IgG (anti-CHKV IFA IgM and IgG by Euroimmun, Lübeck, Germany); (v) the determination of DENV RNA and ZIKV RNA in plasma, saliva, urine, amniotic fluid, and fetal blood using both a pan-flavivirus heminested RT-PCR targeting a conserved region of the NS5 gene [18], as well as virus-specific real-time RT-PCRs, targeting a conserved region in 3′UTR of DENV 1–4 [19] and a portion of the envelope protein gene of ZIKV [20]; and (vi) sequencing of positive pan-flavivirus amplicons.

Advanced diagnosis included the following: (i) NS1 blockade-of-binding (BOB) ELISA assay, in which a human ZIKV-specific anti-NS1 mAb (ZKA35) was labeled with biotin and used in an ELISA based on solid-phase ZIKV NS1. In this assay, plasma or serum samples are diluted (1:10) and incubated on ZIKV NS1-coated plates, followed by the addition of the biotinylated ZKA35 mAb, which detects the presence of ZIKV-specific serum antibodies capable of inhibiting its binding to ZIKV NS1. The ratio or percentage of inhibition were calculated respectively as follows: (1 − [(OD sample − OD neg ctr)/(OD pos ctr − OD neg ctr)]) or (1 − [(OD sample − OD neg ctr)/(OD pos ctr − OD neg ctr)]) × 100 [21], and a cut-off of ratio of 0.36 was calculated. Values lower than 0.36 were specific for ZIKV infection; (ii) ELISpot assay for DENV using peptide pools, 15–18 amino acids in length with an 11–12 amino acid overlap, as antigens. Pool of NS3 peptide from Dengue 1, Dengue 2, Dengue 3, and Dengue 4 were used as stimuli, while human IFN-γ release was determined by ELISpot kits (Diaclone, Besancon, France) as described [22,23]. The mean number of spots from duplicates was adjusted to 1 × 10^6^ PBMC. The net spots per million PBMC was calculated by subtracting the number of spots in response to negative control from the number of spots in response to the corresponding antigen; (iii) ELISpot assay for ZIKV using freeze-dried peptides of 15 amino acids with 11 overlapping of the proteins E (146 peptides), M (49 peptides), and NS1 (166 peptides) from ZIKV (JPT Peptide Technologies, Berlin, Germany). The mean number of spots from duplicate was adjusted to 1 × 10^6^ PBMC. The net spots per million PBMC was calculated by subtracting the number of spots in response to negative control from the number of spots in response to the corresponding antigen. A short-period stimulation, using standard ELISpot assay, allows the quantification of effector memory T cells [24].

Fetal ultrasonography was performed according to International Society of Ultrasound in Obstetrics and Gynecology (ISUOG) Interim Zika Guidance [25]. At each scan, fetal biometric parameters were measured; fetal anatomy was assessed; and, from the 20 weeks’ gestation, a detailed neurosonographic examination was carried out, following ISUOG guidelines [26]. In addition, fetal brain magnetic resonance was performed.

## 3. Results

The results obtained by conventional and advanced assays for the diagnosis of ZIKV infection in the five pregnant women are summarized in Table 1.

### 3.1. Patient 1: Absence of ZIKV Transmission in DENV-Experienced Mother

**Figure 1:**
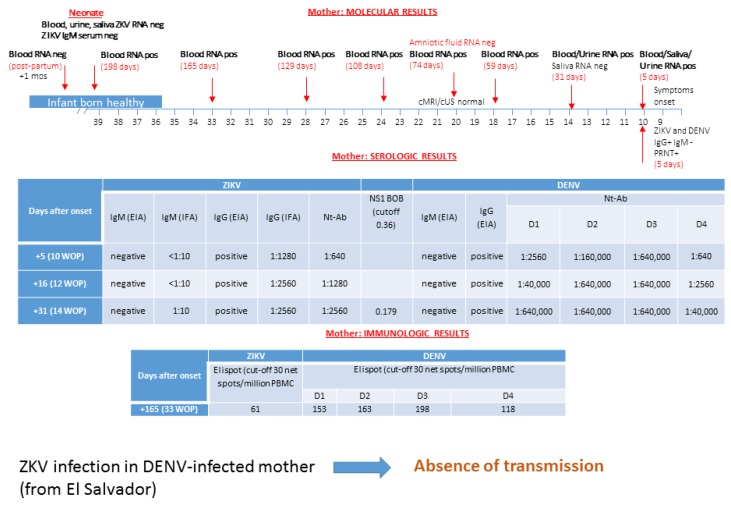
Data on molecular, serological, and immunological diagnosis for Zika virus (ZIKV) and Dengue virus (DENV) infection in a pregnant woman from El Salvador are shown. ZIKV infection in the DENV-infected mother was documented and transmission was not reported. cMRI/cUS: cerebral magnetic resonance imaging/cerebral ultrasonography; PRNT: plaque reduction neutralization test; WOP: week of pregnancy; EIA: enzyme immunoassay; IFA: immunofluorescence assay; Nt-Ab; neutralizing antibodies. In the box of molecular diagnosis, black numbers specify the week of pregnancy, while red numbers in brackets indicate the exact day of gestation. The blockade-of-binding (BOB) assay result is expressed as the ratio of inhibition, while the ELISpot assay is measured as net spots/million PBMC.

A 41-year-old Salvadoran woman living in Italy since 2005 was on holiday in El Salvador with her husband from 11 January to 12 February 2016. She had her last menstrual period on 5 December and she discovered that she was pregnant when she arrived in El Salvador. On 13 February, one day after her arrival in Italy, she presented with maculopapular facial rash, generalized hitching, cough, and diffuse arthromyalgias, which lasted 3–4 days. She recalled being bitten by mosquitoes and heard about ZIKV infection in her native country and was subsequently referred to hospital. On 18 February, blood, saliva, and urine samples were collected and tested for ZIKV, Chikungunya (CHKV), and Dengue (DENV). As shown in Figure 1, at five days after onset, DENV IgG and ZIKV IgG were positive, but specific IgM scored both negative. Neutralizing-antibody titers were also determined to show a significant response to ZIKV and DENV 1–4. Monitoring of serologic response performed two and four weeks after onset showed a four-fold neutralizing-antibody titer increase for ZIKV and DENV 1, 2, and 4. Interestingly, a transient ZIKV IgM antibody response appeared only one month after onset by immunofluorescence assay, and then disappeared at follow-up. ZIKV NS1 blockade-of-binding assay was performed at 31 days after clinical onset and allowed to discriminate ZIKV specific antibodies within a positive IgG response against ZIKV and DENV, as detected by the conventional ELISA assay.

IgG and IgM antibodies against CHKV were negative. On the contrary, ZIKV RNA was simultaneously positive in plasma, urine, and saliva. Sequencing of PCR amplicons showed identity to Central American epidemic strains of ZIKV. DENV RNA was negative in the three maternal samples tested in the acute phase of infection. A diagnosis of acute ZIKV infection at nine weeks of pregnancy was done. Saliva tested positive for ZIKV RNA up to 16 days post-infection. ZIKV RNA detection in urine scored positive at 30 days post-infection. On the contrary, maternal ZIKV RNA in plasma was consistently positive up to 18 week of gestation (59 days post-infection).

Fetal ultrasonography was performed at 12, 14, 16, 18, and 20 weeks of gestation and no signs of microcephaly and intracranial or extracranial abnormalities were observed, apart for a mild increased amount of cerebrospinal fluid around subarachnoid spaces, evidenced from the 28 weeks of gestation.

At 20 weeks of pregnancy, fetal ZIKV infection was assessed by amniocentesis and funiculocentesis. ZIKV RNA detection in amniotic fluid and fetal blood was negative, and specific IgM in fetal plasma were negative as well. Fetal cerebral magnetic reasonance imaging (cMRI) performed did not show any pathological findings. Therefore, ZIKV fetal infection was ruled out at the prenatal investigation.

The patient decided to continue the pregnancy. Fetal ultrasonography and cMRI were repeated at 30 weeks of gestation, and intracranial anatomy was normal as well.

At and after amniocentesis, ZIKV RNA was monitored in maternal blood and prolonged positivity in plasma was detected up to 38 weeks of gestation. She delivered, by cesarean section, a healthy baby at 39 weeks of gestation. Neonatal plasma, urine, and saliva were negative for ZIKV RNA and ZIKV IgM. ZIKV congenital infection was definitively ruled out at birth.

One month after delivery, maternal ZIKV RNA scored negative. ELISpot assay was performed at 33 weeks of gestation, showing a positive specific response for both ZIKV and DENV.

### 3.2. Patient 2: Absence of ZIKV Transmission in DENV-Experienced Mother

**Figure 2:**
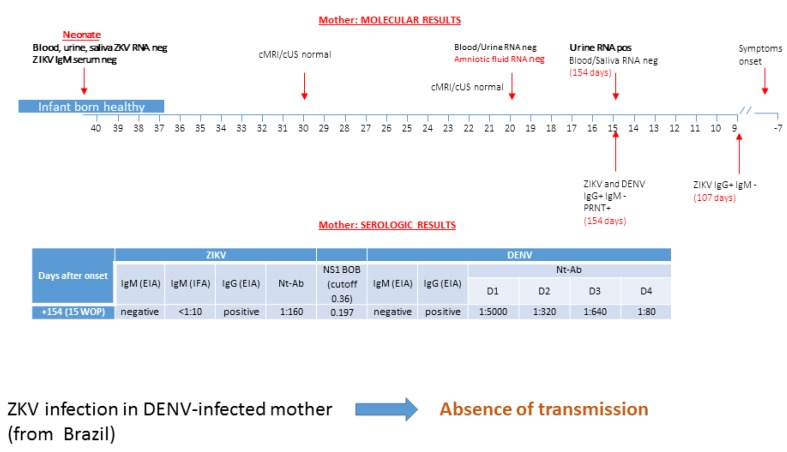
Molecular and serological diagnosis of ZIKV infection performed in a DENV-infected pregnant woman from Brazil is reported. No trasmission was reported. cMRI/cUS: cerebral magnetic resonance imaging/cerebral ultrasonography; PRNT: plaque reduction neutralization test; WOP: week of pregnancy; EIA: enzyme immunoassay; IFA: immunofluorescence assay; Nt-Ab; neutralizing antibodies. In the box of molecular diagnosis, black numbers specify the week of pregnancy, while red numbers in brackets indicate the exact day of gestation. BOB assay result is expressed as the ratio of inhibition.

A native 26 year-old Brazilian woman reported symptoms of maculo-papular rash, arthralgia, and asthenia at approximately seven weeks before pregnancy in Brazil. She underwent in loco ZIKV serologic testing at eight weeks of pregnancy (107 days after onset symptoms). ZIKV IgG testing was positive with specific IgM negative. As her husband was from Lombardy, Italy, the couple decided to refer to our hospital in Pavia for further diagnostic investigation and counseling. She was screened for ZIKV and other flavivirus. At 15 weeks of pregnancy (154 days after onset), a urine sample was positive for ZIKV RNA, while plasma and saliva were negative (Figure 2). The mother showed positive ZIKV and DENV IgG and negative ZIKV and DENV IgM, and virus-neutralizing antibodies positive at a higher titer for DENV 1 with respect to ZIKV NS1 blockade-of-binding assay were able to discriminate ZIKV from DENV infection. Unfortunately, ELISpot assay for DENV and ZIKV had not yet been developed at that time. Neurosonography performed at 20 weeks of pregnancy did not show fetal brain abnormalities. ZIKV RNA was not detected in amniotic fluid, and additional maternal plasma and urine collected for testing were negative as well. At 28 weeks of gestation, fetal cMRI was performed in Brazil and no cerebral malformations were observed. A healthy baby was delivered and absence of ZIKV congenital infection was confirmed at birth (Figure 2).

### 3.3. Patient 3: Absence of ZIKV Transmission in DENV-Experienced Mother

**Figure 3:**
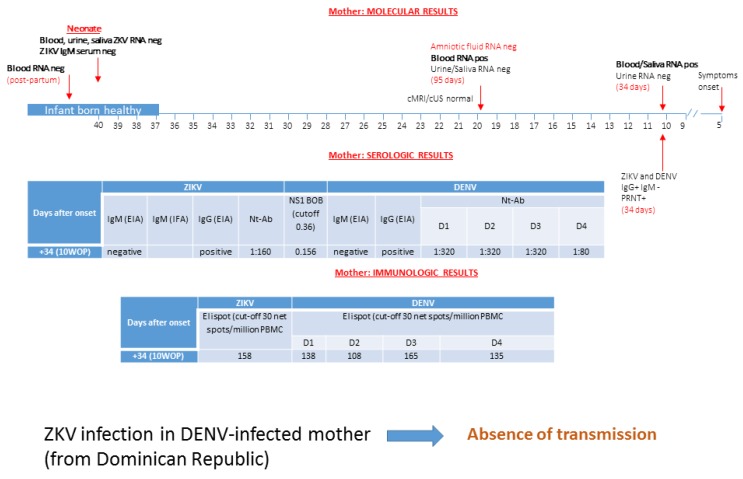
Diagnosis by molecular, serological, and immunological assays of ZIKV infection in a DENV-infected pregnant woman from Brazil was performed. No transmission was reported. cMRI/cUS: cerebral magnetic resonance imaging/cerebral ultrasonography; PRNT: plaque reduction neutralization test; WOP: week of pregnancy; EIA: enzyme immunoassay; IFA: immunofluorescence assay; Nt-Ab; neutralizing antibodies. In the box of molecular diagnosis, black numbers specify the week of pregnancy, while red numbers in brackets indicate the exact day of gestation. BOB assay result is expressed as the ratio of inhibition, while ELISpot assay is measured as net spots/million PBMC.

A 23 year-old woman living in Italy since 2007 was on holiday in her native Dominican Republic in February 2016. She reported fever and generalized rash when she was pregnant at five weeks of gestation. Her husband was concomitantly reporting headache and arthralgia. Five days after her arrival in Italy (34 days after the onset of illness), she was referred to hospital for suspected ZIKV infection. Serologic analysis performed at 10 weeks of gestation documented positive IgG and negative IgM against DENV and ZIKV with neutralizing antibodies positive at significant titer for DENV 1–3 and ZIKV (Figure 3). Blockade-of-binding assay tested positive and was able to distinguish ZIKV from DENV infection. ELISpot assay showed a positive dual response for both ZIKV and DENV. ZIKV RNA was detected in plasma and saliva, but was negative in urine.

Amniocentesis and cordocentesis were performed at 19 weeks of gestation and additional maternal samples were collected (95 days after symptoms onset). ZIKV RNA was detected in plasma, but not in saliva and urine. Amniotic fluid and fetal blood tested negative for ZIKV RNA. Fetal ZIKV IgM were negative as well. Neurosonography and cMRI examination did not show fetal cerebral and/or extra-cerebral abnormalities. She decided to continue the pregnancy, but refused a further cMRI at 30 weeks of gestation, as suggested. She delivered, by cesarean section, at 37 weeks of gestation. Plasma, saliva, and urine of the neonate were negative for ZIKV RNA and specific IgM as well. Postnatal cerebral ultrasonographic examination was normal and congenital ZIKV infection was definitively excluded. Post-partum ZIKV RNA in plasma scored negative.

### 3.4. Patient 4: ZIKV Transmission in DENV-Naive Mother

**Figure 4:**
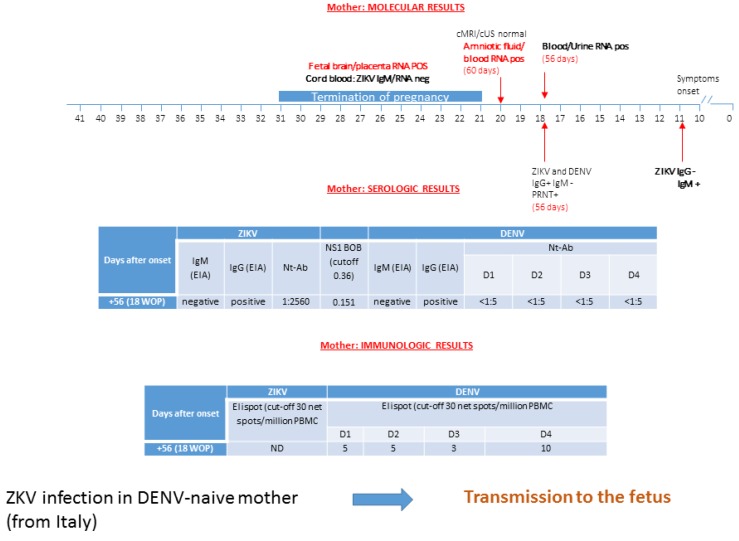
ZIKV infection in a DENV-naïve mother is reported. Diagnosis was performed using molecular, serological, and immunological assays, and transmission to the fetus was reported. cMRI/cUS: cerebral magnetic resonance imaging/cerebral ultrasonography; PRNT: plaque reduction neutralization test; WOP: week of pregnancy; EIA: enzyme immunoassay; IFA: immunofluorescence assay; Nt-Ab; neutralizing antibodies. ND: not done. In the box of molecular diagnosis, black numbers specify the week of pregnancy, while red numbers in brackets indicate the exact day of gestation. BOB assay result is expressed as the ratio of inhibition, while ELISpot assay is measured as net spots/million PBMC.

An Italian woman living in the Dominican Republic since 2011 had a self-limited itchy generalized maculo-papular rash at 10 weeks of gestation. Serologic investigation for suspected ZIKV infection was performed locally, showing positive IgM and negative IgG against ZIKV at four days after symptoms onset. At 18 weeks of pregnancy, she returned to Italy and was referred to our hospital. At 56 days after onset, molecular testing found ZIKV RNA in plasma and urine, while in saliva, it scored negative (Figure 4). Serological results for both ZIKV and DENV IgG were positive, while results for both ZIKV and DENV IgM were negative. In terms of virus-neutralizing antibodies, we observed a significant positive titer for ZIKV in absence of neutralizing antibodies for DENV 1–4.

Blockade-of-binding assay excluded DENV infection. ELISpot investigation could be performed for DENV only, showing a negative cellular-immune response. At 19 weeks of gestation (or 60 days after onset), amniocentesis was performed and ZIKV RNA was found both in amniotic fluid and maternal plasma. Neurosonography showed the presence of a small intracranial calcification, but fetal cerebral cMRI was normal. The patient elected to terminate her pregnancy at 21 weeks of gestation. Conclusive evidence of fetal infection came from the presence of ZIKV RNA in brain tissue (cortex) and placenta, but not in umbilical cord, fetal skin, lung, heart, liver, kidney, cerebellum, eye, adrenal gland, and thymus.

### 3.5. Patient 5: ZIKV Transmission in DENV-Naive Mother

An Italian male infant at five months of age came to the clinic for evaluation. His birth weight was 5.6 kg (<3° centile), height was 52 cm (<3° centile), and cranial circumference was 32 cm (<3° centile). The infant had craniofacial disproportion, arthrogryposis, dysphagia, convergent strabismus, and no eye contact without chorioretinal abnormalities, normal hearing evaluations, hypertonia, pyramidal, and extrapyramidal signs with dystonic movements consistent with mixed cerebral palsy and epilepsy (likely related to the cortical malformations). Neuroimaging (CT scan without contrast) showed bilateral diffuse malformations of cortical development, brainstem hypoplasia, and calcifications, predominantly in the subcortical and basal ganglia. All neuroimaging showed evidence of decreased brain volume with ventriculomegaly. The infant was too young to be adequately assessed for cognitive deficits.

**Figure 5:**
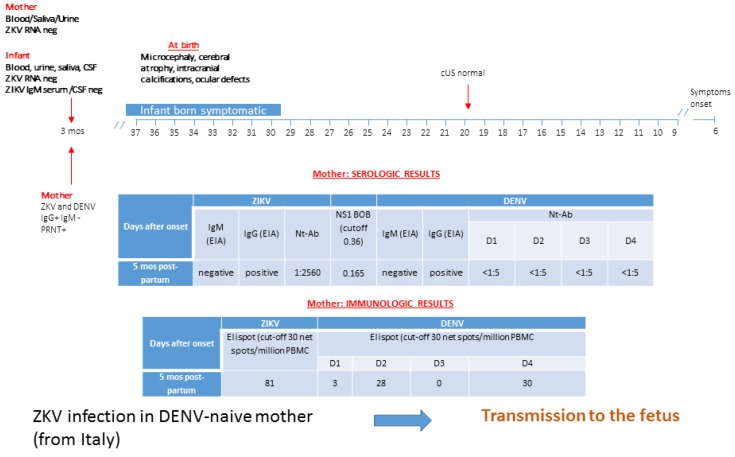
ZIKV infection in a DENV-naïve mother is reported. Diagnosis was performed using molecular, serological, and immunological assays, and transmission to the fetus was reported. cMRI/cUS: cerebral magnetic resonance imaging/cerebral ultrasonography; PRNT: plaque reduction neutralization test; WOP: week of pregnancy; EIA: enzyme immunoassay; IFA: immunofluorescence assay; Nt-Ab; neutralizing antibodies. In the box of molecular diagnosis, black numbers specify the week of pregnancy, while red numbers in brackets indicate the exact day of gestation. BOB assay result is expressed as the ratio of inhibition, while ELISpot assay is measured as net spots/million PBMC.

He was born in the Dominican Republic, where the Italian mother had been living since 2015. At six weeks of gestation, she had fever, headache, and macular rash, but no serologic testing for ZIKV infection was performed. Fetal cerebral ultrasonography (cUS) investigation was normal during pregnancy, but a microcephalic baby was delivered at 37 weeks of gestation (Figure 5). At first referral in Italy (five months post-partum), serologic testing showed positive IgG and negative IgM for ZIKV and DENV. Virus-neutralizing antibodies were positive at significant titer for ZIKV and negative for DENV 1–4. Blockade-of-binding assay tested positive for ZIKV-specific antibodies, while DENV infection was ruled out. ELISpot investigation was performed for DENV and ZIKV, showing a negative T-cell response for DENV, while it was positive for ZIKV. Blood, urine, and saliva scored negative for both DENV and ZIKV RNA.

At five months of age, ZIKV and DENV RNA were negative in blood, saliva, urine, and CSF, and IgM was negative in serum and CSF. No cross-reactivity with DENV-specific IgM was seen in the samples tested. Negative laboratory test results for infectious causes of congenital microcephaly (toxoplasmosis, cytomegalovirus, rubella) were obtained. IgG tested positive for both ZIKV and DENV, but blockade-of binding assay showed inconclusive results. Therefore, laboratory evidence of congenital Zika virus infection could not be confirmed at five months of age. Persistence of ZIKV-specific antibodies at 6–9 months post-infection was assessed to confirm clinical diagnosis of congenital ZIKV infection.

## 4. Discussion

When the World Health Organization (WHO) declared an international public health state of emergency in February 2016, the Italian Ministry of Health issued a guideline for all professionals on how to handle a confirmed or inconclusive laboratory testing for ZIKV in pregnancy. In particular, management of pregnant women included ultrasonographic investigation and, in the case of pathological findings, amniocentesis to investigate genetic disorders and congenital infections, including ZIKV. Growing evidence suggests that ultrasound abnormalities start to be detectable from 18th to 20th week of pregnancy. Because, in Italy, voluntary abortion is allowed up to the 22nd week of gestation, a tailored management of ZIKV infection was adopted in our center according to the trimester of infection.

Several studies in which the foetus or neonate had congenital abnormalities suggested that the most likely period of exposure to ZIKV was during the first or early second trimester [27,28]. For this reason, amniocentesis was considered for ZIKV infections of the first trimester, and was performed at the 20th week of gestation, in association with fetal cUS and cMRI, being aware that ZIKV can be detected in amniotic fluid, but a negative result at the 20th week of gestation does not rule out the diagnosis [29,30].

According to the CDC algorithm [31], it became evident that diagnosis of ZIKV infection in the mother should rely on the detection of viral RNA, because cross-reactivity (IgG, IgM, and Nt-Abs) with other flavivirus family members is confounding. Travel or residence in endemic or epidemic areas were a key point, but country of origin also needed to be taken into account for the interpretation of serological results. In our five pregnant women, three of them travelled to areas where ZIKV was active, and the remaining two were autochthonous infections. However, two travelers (patient 1 and 3) and one autochthonous (patient 2) were native from Latin America, where there is a co-circulation of ZIKV and DENV and yellow fever (YF) vaccination is extensive. The remaining two (patient 4 and 5) were from Italy, where DENV is not endemic. Patients 1–3 were vaccinated for YF, and thus, as reported by other authors, a lower risk of developing microcephaly may be posed to the offspring of pregnant women in regions with high YF vaccination coverage [32]. All patients experienced symptoms that were considered at the exact time of exposure. In native patients (1–3), IgM were undetectable at first referral (5, 154, and 34 days after onset, respectively). This pattern of antibody response characterized by the absence or blunted IgM response at follow-up has been described in the literature both in patients with heterologous DENV infection [33] and in patients with ZIKV infection and previous DENV immunity [34]. In the context of sequential flavivirus infections, IgM levels are strongly reduced to non-detectable levels [35].

The high cross-reactivity between flaviviruses represents a crucial issue in terms of serological diagnosis in DENV-immune ZIKV-infected patients, especially in pregnant women. Therefore, we set up a new antibody-based assay able to discriminate ZIKV from DENV infection [21]. This assay is a competition ELISA based on NS1 blockade-of-binding (BOB) using a ZIKV NS1-specific human monoclonal antibody generated in vitro from a B cell clone obtained from our ZIKV patient #1 [36]. In our pregnant women, ZIKV and DENV IgG scored positive and ZIKV specific antibodies could be confirmed by NS1 BOB assay. Therefore, it was crucial for the diagnosis of ZIKV infection in pregnant women living in areas exposed to ZIKV and endemic for other flaviviruses. PRNT was performed according to CDC guidelines. Nt-Abs were positive for ZIKV and DENV (patients 1–3) and titer was higher for DENV than for ZIKV in all the three patients. For these unclear results, we set up a new immunological method for the characterization of both ZIKV and DENV-specific T-cell response in both native and non-native pregnant women. We observed that ZIKV and DENV-specific T-cell response were both positive in native pregnant women (patient#1 and #3), while in a non-native pregnant woman (patient #5), ZIKV-specific response was reported in the absence of DENV-specific T-cell response. According to our previous results, DENV-specific T-cell response is almost undetectable in DENV-naïve patients with acute ZIKV infection, while a significant and specific T-cell response to DENV can be detected in DENV-immune patients with acute ZIKV infection [37]. Focusing on the characterization of ZIKV-specific T-cell response, we observed that, in pregnant women who experienced previous DENV infection, T-cell response to M protein was almost undetectable, while NS1- and E-specific T-cell responses were higher. We reported a detectable M-specific T-cell response only in the DENV-naïve pregnant woman (patient 5). Other studies reported that ZIKV-specific CD8 T cell response is predominantly targeted on structural protein such as E, prM, and C [38]. The role of different DENV- and ZIKV epitope-specific T-cell responses in determining susceptibility to vertical transmission has to be further dissected.

The conventional serological approach is recommended by CDC guidelines [39] to rule out potential false-positive results. However, in 3/3 native patients (100%), PRNT did not allow to confirm or exclude ZIKV infection. Therefore, in these three cases, advanced serology (NS1 BOB assay) was mandatory to discriminate ZIKV infection from DENV. In patient 4 (non-native), IgM were positive five days after onset, but at first referral in Italy, IgM were negative (56 days post-infection). Therefore, diagnosis could not be reliably excluded by undetectable IgM. Even in the two non-native patients, IgG cross-reactivity with DENV was reported, but in both cases, a high neutralizing antibody titer for ZIKV, but not for DENV, was shown. NS1 BOB assay confirmed the presence of specific antibodies reactive to ZIKV only.

Current CDC guidelines recommend testing serum or urine by RT-PCR within 14 days of symptoms or last potential exposure [39,40]. In our five pregnant women, ZIKV RNA was detected after onset of symptoms up to 34 days in saliva, 154 days in urine, and 198 days in plasma. Long periods of viremia have been observed during pregnancy in the presence of a fetus infected with ZIKV [27]. It is unclear whether this persistent viremia results from an infected fetus or placenta, or was a consequence of changes in the maternal immune system during pregnancy. As ZIKV targets different primary human placental cells [41], it may be speculated that placenta rather than fetal infection might be the source of prolonged viremia.

The congenital ZIKV syndrome portends a constellation of findings, inclusive of intracranial abnormalities, which may include microcephaly. However, patient 4 is a clear demonstration of the limitations of findings by ultrasonography alone for the evaluation of congenital ZIKV infection. Sonographic screening could be complementary, while neurosonography should be used in parallel with molecular diagnostics, irrespective of the presence or absence of microcephaly, because fetal brain malformations may be either incident to or causative of microcephaly. Fetal cMRI can also be performed, as an adjunct to neurosonology, to better characterize intracranial abnormalities. In patient 4, ZIKV RT-PCR assays were positive only in fetal brain tissues at autopsy without abnormalities at cUS and cMRI. However, a cerebral calcification was seen during neurosonology, suggesting that the neurological damage caused by ZIKV is a continuum that may begin in utero, but does not necessarily end with delivery. Sequence analysis showed 99–100% identity with Zika virus strains isolated from Brazil during 2015. The patterns of injury are likely to follow from both cellular injury at the time of infection as well as subsequent damage as the brain develops [42].

In our pregnant woman with a pre-existing DENV immunity, DENV Nt-Abs and DENV specific T-cells stated for multiple past DENV infections. In this setting, it was proposed that ZIKV could be considered as a fifth DENV serotype owing to the 77% sequence homology between epitopes [38]. Indeed, ZIKV E protein shares a high degree of homology with DENV E protein and the fusion loop was described as a target for broadly cross-reactive antibodies against DENV, as well as other flaviviruses [43]. Another target of significant interest is the envelope dimer epitope (EDE), which induces a broadly neutralizing antibody response; it has been described that EDE-specific human mAbs derived from memory B cells of DENV-infected patients cross-neutralized ZIKV. Because ZIKV outbreaks are largely localized in DENV-endemic areas, the role of pre-existing DENV-induced antibodies in enhancing ZIKV infection is a matter of concern. It has been hypothesized that antibody-dependent enhancement (ADE) contributes to the increased disease severity. On the other hand, in one study, EDE mAbs potently neutralized all four DENV serotypes as well as ZIKV, suggesting that the neutralization potential of antibodies targeting certain epitopes, such as EDE, may block the ADE effect [44]. It has been demonstrated that sera from patients with secondary DENV infection can strongly neutralize ZIKV, while sera from patients with primary DENV infection exhibit limited cross-neutralizing activity [12]. In vivo studies showed that, after secondary DENV infection, individuals develop multitypic Nt-Abs, not only against the exposed serotype, but also against serotypes to which they have not yet been exposed [45]. Such heterotypic Nt-Abs contribute to protection against the non-experienced serotypes during a third or fourth DENV infection, as suggested by the lower rates of hospital admissions [46] and reduced risk of symptomatic DENV infection in humans [47]. In this sense, prior DENV infection might be a cofactor that could affect the risk of transmission and adverse birth outcomes following ZIKV infection during pregnancy. Given the overlapping presence of DENV in the majority of ZIKV epidemic regions, there is a pressing need to better understand the extent and characteristics of DENV–ZIKV immunological cross-reactivity and cross-protection.

The results shown in this paper underlined the complex serological interaction between DENV and ZIKV. In detail, we described three cases of ZIKV infection in DENV-immune mothers (patients 1, 2, and 3) with the absence of transmission to the fetus. In these women, pre-existing DENV infection was confirmed by specific ELISpot assay and ZIKV infection was diagnosed by NS1 BOB assay. On the other side, we also described two cases (patients 4 and 5) of ZIKV infection in DENV-naïve mothers; in both cases, ZIKV transmission to the fetus was reported. In these two women, previous DENV infection was excluded by both PRNT and ELISpot assay, while the presence of specific ZIKV antibodies was confirmed by NS1 BOB ELISA.

Despite being limited in number, our clinical cases seem to support the idea that, in experienced populations, DENV infection may be protective from ZIKV transmission to the fetus. DENV-specific effector memory T cells, called protective memory T cells, and characterized by rapid effector function in response to antigenic stimulation, were actually demonstrated in ZIKV non-transmitter mothers (patients 1 and 3). On the contrary, in transmitter mothers, DENV effector memory T-cells were absent (patients 4 and 5). Although this topic needs to be further investigated, the higher estimated prevalence of the ZIKV-associated microcephaly among live births in USA and Colombia, as compared with Brazil, where DENV is endemic, offers an epidemiologic acknowledgement to this mechanism of protection [8].

Regarding the problem of ZIKV-associated microcephaly before 2016, papers reported retrospective data on this association during the epidemic in French Polynesia. After the South America pandemic, maybe because of the large immunization of the population, the rate of ZIKV-associated microcephaly decreased significantly [48].

In conclusion, the current ZIKV diagnostic capabilities include ZIKV RNA detection and IgM serology, with a heavy reliance on PRNT for confirmation. However, missing elements include IgG interpretation in DENV-experienced patients and cellular immunity testing for both ZIKV and DENV. To dissect ZIKV-specific and DENV-specific T-cell effector memory and epitope-specific response in DENV-naïve and DENV-experienced patients is warranted for understanding protection and pathology in ZIKV-infected pregnant women. 

## Figures and Tables

**Table 1:** Characteristics of all pregnant women.

Characteristics	Patient #1							Patient #2		Patient #3		Patient #4			Patient #5
days after symptoms onset	5	16	31	59	74	165	198	107	154	34	95	11	56	60	3 months post-partum
ZIKV RNA in blood	Pos		Pos	Pos	Pos	Pos	Pos		Neg	Pos	Pos		Pos	Pos	Neg
ZIKV RNA in urine	Pos		Pos	Neg					Pos	Neg	Neg		Pos		Neg
ZIKV RNA in saliva	Pos		Neg	Neg					Neg	Pos	Neg				Neg
ZIKV RNA in AF					Neg						Neg			Pos	
ZIKV IgM EIA	Neg	Neg	Neg					Neg	Neg	Neg		Pos	Neg		Neg
ZIKV IgG EIA	Pos	Pos	Pos					Pos	Pos	Pos		Neg	Pos		Pos
ZIKV NT Abs	1:640	1:1280	1:2560						1:160	1:160			1:2560		1:2560
DENV IgM EIA	Neg	Neg	Neg						Neg	Neg			Neg		Neg
DENV IgG EIA	Pos	Pos	Pos						Pos	Pos			Pos		Pos
DENV NT Abs															
DENV1	1:2560	1:40,000	1:640,000						1:5000	1:320			<5		<5
DENV2	1:160,000	1:640,000	1:640,000						1:320	1:320			<5		<5
DENV 3	1:640,000	1:640,000	1:640,000						1:640	1:320			<5		<5
DENV 4	1:640	1:2560	1:640,000						1:80	1:80			<5		<5
BOB assay *			0.179						0.197	0.156			0.151		0.165
ZIKV ELISPOT **						61									81
DENV ELISPOT **															
DENV1						153							5		3
DENV2						163							5		28
DENV 3						198							3		0
DENV 4						118							10		30
outcome	no transmission							no transmission		no transmission		transmission			transmission

* Blockade-of-binding (BOB) assay: cut off 0.36 (confirmed Zika virus (ZIKV) Ab < 0.36), all results are expressed in ratio; ** responses are given as net spots/million PBMC, cut-off of response 30 net spots/million PBMC; EIA: enzyme immunoassay; AF: amniotic fluid; NT Abs: neutralizing antibodies; results of NT Abs < 5 are considered negative. DENV, Dengue virus.

## Abbreviations

ZIKV	Zika virus
DENV	Dengue virus
NS1 BOB	blockade-of-binding assay
YF	yellow fever
cMRI/cUS	cerebral magnetic reasonance imaging/cerebral ultrasonography
PRNT	plaque reduction neutralization test
Nt-Abs	neutralizing antibodies
EDE	envelope dimer epitope
ADE	antibody-dependent enhancement
